# Supplementary material for: Meta-Analysis and Gene Set Enrichment Relative to ER Status Reveal Elevated Activity of MYC and E2F in the “Basal” Breast Cancer Subgroup
Source: PLoS One. 2009 Mar 9;4(3):e4710. doi: 10.1371/journal.pone.0004710 (PMC2650420; doi:10.1371/journal.pone.0004710)
Supplement: Dataset S1 — GSEA results from first screen. The file contains a zipped archive of the HTML results of the second GSEA screen. Reports for enriched or depleted datasets may be accessed from the “index.html” link within the my_analysis.GseaPreranked.1219975950625.rpt.zip ZIP archive. Enrichment plots are available for each of the 50 most enriched and depleted gene sets. (13.32 MB ZIP) [file pone.0004710.s001.zip › my_analysis.GseaPreranked.1219975950625.rpt.zip!//CHR11Q13.html]

Details for gene set CHR11Q13[GSEA]

|  || Dataset | zSco.ERneg.to.ERpos\_collapsed |
| Phenotype | NoPhenotypeAvailable |
| Upregulated in class | na\_neg |
| GeneSet | CHR11Q13 |
| Enrichment Score (ES) | -0.42029363 |
| Normalized Enrichment Score (NES) | -2.0818188 |
| Nominal p-value | 0.0 |
| FDR q-value | 0.004645417 |
| FWER p-Value | 0.081 |
Table: GSEA Results Summary

  

Fig 1: Enrichment plot: CHR11Q13      
 Profile of the Running ES Score & Positions of GeneSet Members on the Rank Ordered List

  

| PROBE | GENE SYMBOL | GENE\_TITLE | RANK IN GENE LIST | RANK METRIC SCORE | RUNNING ES | CORE ENRICHMENT || 1 | GSTP1 |  |  | 42 | 6.388 | 0.0144 | No |
| 2 | GAL |  |  | 336 | 4.836 | 0.0054 | No |
| 3 | PPP1R14B |  |  | 522 | 4.303 | 0.0032 | No |
| 4 | CLPB |  |  | 681 | 3.971 | 0.0021 | No |
| 5 | KCTD14 |  |  | 690 | 3.950 | 0.0123 | No |
| 6 | WNT11 |  |  | 710 | 3.913 | 0.0217 | No |
| 7 | FOLR1 |  |  | 800 | 3.774 | 0.0253 | No |
| 8 | DHCR7 |  |  | 1101 | 3.358 | 0.0117 | No |
| 9 | P2RY6 |  |  | 1182 | 3.259 | 0.0146 | No |
| 10 | CTSW |  |  | 1662 | 2.792 | -0.0142 | No |
| 11 | ZFPL1 |  |  | 1667 | 2.787 | -0.0069 | No |
| 12 | MRPL11 |  |  | 1693 | 2.764 | -0.0011 | No |
| 13 | SPTBN2 |  |  | 1772 | 2.712 | 0.0004 | No |
| 14 | SERPINH1 |  |  | 1861 | 2.639 | 0.0009 | No |
| 15 | RASGRP2 |  |  | 2003 | 2.527 | -0.0028 | No |
| 16 | MYO7A |  |  | 2059 | 2.477 | -0.0002 | No |
| 17 | ADRBK1 |  |  | 2257 | 2.345 | -0.0087 | No |
| 18 | ARL2 |  |  | 2296 | 2.323 | -0.0052 | No |
| 19 | POLA2 |  |  | 2649 | 2.117 | -0.0262 | No |
| 20 | MGC3032 |  |  | 2723 | 2.079 | -0.0260 | No |
| 21 | EHD1 |  |  | 2779 | 2.053 | -0.0246 | No |
| 22 | SLC22A11 |  |  | 2782 | 2.051 | -0.0191 | No |
| 23 | NEU3 |  |  | 3032 | 1.917 | -0.0328 | No |
| 24 | FOSL1 |  |  | 3184 | 1.847 | -0.0392 | No |
| 25 | FOLR3 |  |  | 3215 | 1.831 | -0.0364 | No |
| 26 | MOGAT2 |  |  | 3350 | 1.772 | -0.0417 | No |
| 27 | LOC399904 |  |  | 3427 | 1.738 | -0.0427 | No |
| 28 | GDPD5 |  |  | 3524 | 1.698 | -0.0454 | No |
| 29 | DNAJC4 |  |  | 3691 | 1.622 | -0.0535 | No |
| 30 | FOLR2 |  |  | 3714 | 1.616 | -0.0508 | No |
| 31 | C11ORF24 |  |  | 3831 | 1.578 | -0.0552 | No |
| 32 | SLCO2B1 |  |  | 3839 | 1.574 | -0.0514 | No |
| 33 | MARK2 |  |  | 3844 | 1.573 | -0.0474 | No |
| 34 | RCE1 |  |  | 4317 | 1.383 | -0.0795 | No |
| 35 | PTPRCAP |  |  | 4392 | 1.359 | -0.0814 | No |
| 36 | SART1 |  |  | 4463 | 1.332 | -0.0831 | No |
| 37 | FLJ33790 |  |  | 4530 | 1.308 | -0.0845 | No |
| 38 | CCDC85B |  |  | 4814 | 1.218 | -0.1027 | No |
| 39 | YAP1 |  |  | 4988 | 1.160 | -0.1127 | No |
| 40 | KCNK7 |  |  | 5051 | 1.136 | -0.1143 | No |
| 41 | FKSG44 |  |  | 5052 | 1.136 | -0.1111 | No |
| 42 | FGF4 |  |  | 5081 | 1.122 | -0.1102 | No |
| 43 | CABP2 |  |  | 5228 | 1.073 | -0.1183 | No |
| 44 | NDUFV1 |  |  | 5233 | 1.070 | -0.1157 | No |
| 45 | GIF |  |  | 5256 | 1.062 | -0.1145 | No |
| 46 | SHANK2 |  |  | 5322 | 1.037 | -0.1165 | No |
| 47 | SAC3D1 |  |  | 5364 | 1.023 | -0.1169 | No |
| 48 | PITPNM1 |  |  | 5370 | 1.022 | -0.1144 | No |
| 49 | KRTAP5-8 |  |  | 5456 | 0.992 | -0.1182 | No |
| 50 | FGF3 |  |  | 5537 | 0.962 | -0.1216 | No |
| 51 | MRPL48 |  |  | 5933 | 0.837 | -0.1494 | No |
| 52 | RPS3 |  |  | 5994 | 0.814 | -0.1517 | No |
| 53 | CDC42EP2 |  |  | 6126 | 0.776 | -0.1595 | No |
| 54 | LRP5 |  |  | 6202 | 0.750 | -0.1632 | No |
| 55 | ALDH3B1 |  |  | 6207 | 0.749 | -0.1614 | No |
| 56 | PHOX2A |  |  | 6221 | 0.743 | -0.1604 | No |
| 57 | LRFN4 |  |  | 6297 | 0.717 | -0.1641 | No |
| 58 | KRTAP5-9 |  |  | 6323 | 0.710 | -0.1640 | No |
| 59 | BANF1 |  |  | 6445 | 0.668 | -0.1714 | No |
| 60 | FLJ22596 |  |  | 6448 | 0.667 | -0.1697 | No |
| 61 | TBX10 |  |  | 6524 | 0.642 | -0.1737 | No |
| 62 | STIP1 |  |  | 6584 | 0.625 | -0.1764 | No |
| 63 | MAP3K11 |  |  | 6783 | 0.558 | -0.1900 | No |
| 64 | PACS1 |  |  | 6812 | 0.547 | -0.1906 | No |
| 65 | RAB6A |  |  | 6865 | 0.529 | -0.1931 | No |
| 66 | PRKRIR |  |  | 7150 | 0.436 | -0.2135 | No |
| 67 | LRRC51 |  |  | 7404 | 0.345 | -0.2318 | No |
| 68 | CD5 |  |  | 7509 | 0.304 | -0.2389 | No |
| 69 | CHKA |  |  | 7547 | 0.291 | -0.2409 | No |
| 70 | UNC93B1 |  |  | 7653 | 0.247 | -0.2482 | No |
| 71 | SCGB1D1 |  |  | 7680 | 0.238 | -0.2496 | No |
| 72 | NRXN2 |  |  | 7812 | 0.184 | -0.2590 | No |
| 73 | FBXL11 |  |  | 7871 | 0.157 | -0.2630 | No |
| 74 | PLCB3 |  |  | 7937 | 0.133 | -0.2676 | No |
| 75 | PRO1880 |  |  | 8181 | 0.045 | -0.2860 | No |
| 76 | CLCF1 |  |  | 8204 | 0.033 | -0.2875 | No |
| 77 | RELA |  |  | 8275 | 0.008 | -0.2928 | No |
| 78 | ROM1 |  |  | 8483 | -0.071 | -0.3084 | No |
| 79 | ACTN3 |  |  | 8512 | -0.084 | -0.3103 | No |
| 80 | DKFZP547G183 |  |  | 8549 | -0.102 | -0.3128 | No |
| 81 | RPS6KB2 |  |  | 8570 | -0.111 | -0.3140 | No |
| 82 | UCP3 |  |  | 8593 | -0.126 | -0.3153 | No |
| 83 | RIN1 |  |  | 8655 | -0.154 | -0.3195 | No |
| 84 | FAU |  |  | 8728 | -0.181 | -0.3245 | No |
| 85 | PPME1 |  |  | 8840 | -0.227 | -0.3323 | No |
| 86 | CD248 |  |  | 9033 | -0.327 | -0.3461 | No |
| 87 | OVOL1 |  |  | 9106 | -0.365 | -0.3505 | No |
| 88 | SF1 |  |  | 9117 | -0.369 | -0.3503 | No |
| 89 | AIP |  |  | 9211 | -0.409 | -0.3562 | No |
| 90 | TCIRG1 |  |  | 9535 | -0.580 | -0.3792 | No |
| 91 | C11ORF67 |  |  | 9650 | -0.646 | -0.3861 | No |
| 92 | CHCHD8 |  |  | 9794 | -0.721 | -0.3950 | No |
| 93 | NAT11 |  |  | 9798 | -0.722 | -0.3933 | No |
| 94 | MAP4K2 |  |  | 9834 | -0.746 | -0.3939 | No |
| 95 | MEN1 |  |  | 9886 | -0.776 | -0.3956 | No |
| 96 | FCHSD2 |  |  | 9898 | -0.782 | -0.3943 | No |
| 97 | C11ORF51 |  |  | 9907 | -0.789 | -0.3927 | No |
| 98 | CAPN1 |  |  | 9938 | -0.802 | -0.3928 | No |
| 99 | CCDC87 |  |  | 9948 | -0.809 | -0.3913 | No |
| 100 | UVRAG |  |  | 10069 | -0.889 | -0.3980 | No |
| 101 | PPFIA1 |  |  | 10310 | -1.038 | -0.4134 | No |
| 102 | MUS81 |  |  | 10402 | -1.092 | -0.4173 | Yes |
| 103 | SIPA1 |  |  | 10408 | -1.095 | -0.4147 | Yes |
| 104 | EHBP1L1 |  |  | 10458 | -1.127 | -0.4153 | Yes |
| 105 | IGHMBP2 |  |  | 10460 | -1.130 | -0.4122 | Yes |
| 106 | RNF121 |  |  | 10488 | -1.152 | -0.4111 | Yes |
| 107 | CPT1A |  |  | 10511 | -1.172 | -0.4096 | Yes |
| 108 | BRMS1 |  |  | 10515 | -1.175 | -0.4066 | Yes |
| 109 | PDE2A |  |  | 10541 | -1.201 | -0.4052 | Yes |
| 110 | KIAA0280 |  |  | 10587 | -1.234 | -0.4052 | Yes |
| 111 | CTTN |  |  | 10594 | -1.239 | -0.4022 | Yes |
| 112 | C11ORF30 |  |  | 10656 | -1.284 | -0.4034 | Yes |
| 113 | RSF1 |  |  | 10771 | -1.367 | -0.4083 | Yes |
| 114 | NDUFS8 |  |  | 10844 | -1.427 | -0.4098 | Yes |
| 115 | CFL1 |  |  | 10849 | -1.430 | -0.4062 | Yes |
| 116 | ODZ4 |  |  | 10886 | -1.461 | -0.4049 | Yes |
| 117 | POLR2G |  |  | 10905 | -1.477 | -0.4022 | Yes |
| 118 | KIAA0404 |  |  | 10931 | -1.509 | -0.4000 | Yes |
| 119 | DKFZP586P0123 |  |  | 10944 | -1.523 | -0.3967 | Yes |
| 120 | OTUB1 |  |  | 10966 | -1.543 | -0.3940 | Yes |
| 121 | C11ORF2 |  |  | 10980 | -1.551 | -0.3908 | Yes |
| 122 | SF3B2 |  |  | 11028 | -1.592 | -0.3900 | Yes |
| 123 | CST6 |  |  | 11043 | -1.611 | -0.3866 | Yes |
| 124 | GAB2 |  |  | 11090 | -1.656 | -0.3855 | Yes |
| 125 | RBM4 |  |  | 11115 | -1.678 | -0.3827 | Yes |
| 126 | FAM86C |  |  | 11133 | -1.689 | -0.3794 | Yes |
| 127 | SLC29A2 |  |  | 11157 | -1.708 | -0.3764 | Yes |
| 128 | YIF1A |  |  | 11204 | -1.746 | -0.3751 | Yes |
| 129 | RBM4B |  |  | 11290 | -1.815 | -0.3766 | Yes |
| 130 | ZNHIT2 |  |  | 11316 | -1.839 | -0.3734 | Yes |
| 131 | PAK1 |  |  | 11386 | -1.918 | -0.3734 | Yes |
| 132 | RTN3 |  |  | 11481 | -2.013 | -0.3750 | Yes |
| 133 | FTH1 |  |  | 11495 | -2.024 | -0.3704 | Yes |
| 134 | CENTD2 |  |  | 11514 | -2.050 | -0.3662 | Yes |
| 135 | HTATIP |  |  | 11650 | -2.166 | -0.3705 | Yes |
| 136 | EFEMP2 |  |  | 11785 | -2.311 | -0.3743 | Yes |
| 137 | SSSCA1 |  |  | 11818 | -2.347 | -0.3703 | Yes |
| 138 | LRRC54 |  |  | 11842 | -2.362 | -0.3655 | Yes |
| 139 | KLC2 |  |  | 11886 | -2.421 | -0.3622 | Yes |
| 140 | SAPS3 |  |  | 11957 | -2.512 | -0.3606 | Yes |
| 141 | NUMA1 |  |  | 12034 | -2.614 | -0.3592 | Yes |
| 142 | DPF2 |  |  | 12036 | -2.621 | -0.3520 | Yes |
| 143 | MRPL49 |  |  | 12054 | -2.645 | -0.3460 | Yes |
| 144 | DRAP1 |  |  | 12067 | -2.656 | -0.3396 | Yes |
| 145 | ALDH3B2 |  |  | 12102 | -2.708 | -0.3348 | Yes |
| 146 | CDK2AP2 |  |  | 12122 | -2.729 | -0.3287 | Yes |
| 147 | CORO1B |  |  | 12164 | -2.796 | -0.3241 | Yes |
| 148 | B3GNT1 |  |  | 12207 | -2.848 | -0.3195 | Yes |
| 149 | UCP2 |  |  | 12270 | -2.930 | -0.3161 | Yes |
| 150 | LRRC32 |  |  | 12284 | -2.948 | -0.3090 | Yes |
| 151 | SCGB2A1 |  |  | 12325 | -2.994 | -0.3038 | Yes |
| 152 | RAD9A |  |  | 12329 | -2.996 | -0.2958 | Yes |
| 153 | ARRB1 |  |  | 12336 | -3.004 | -0.2880 | Yes |
| 154 | FLJ22531 |  |  | 12337 | -3.006 | -0.2797 | Yes |
| 155 | FADD |  |  | 12367 | -3.048 | -0.2735 | Yes |
| 156 | SPCS2 |  |  | 12377 | -3.059 | -0.2658 | Yes |
| 157 | C11ORF68 |  |  | 12381 | -3.068 | -0.2576 | Yes |
| 158 | ARHGEF17 |  |  | 12465 | -3.206 | -0.2551 | Yes |
| 159 | TM7SF2 |  |  | 12522 | -3.295 | -0.2503 | Yes |
| 160 | ZDHHC24 |  |  | 12546 | -3.334 | -0.2428 | Yes |
| 161 | CCS |  |  | 12616 | -3.459 | -0.2386 | Yes |
| 162 | PCF11 |  |  | 12637 | -3.505 | -0.2304 | Yes |
| 163 | FKBP2 |  |  | 12681 | -3.577 | -0.2239 | Yes |
| 164 | NADSYN1 |  |  | 12731 | -3.691 | -0.2174 | Yes |
| 165 | BAD |  |  | 12744 | -3.724 | -0.2081 | Yes |
| 166 | PPP1CA |  |  | 12748 | -3.737 | -0.1980 | Yes |
| 167 | CTSF |  |  | 12816 | -3.921 | -0.1924 | Yes |
| 168 | MTL5 |  |  | 12830 | -3.952 | -0.1825 | Yes |
| 169 | VEGFB |  |  | 12855 | -4.021 | -0.1732 | Yes |
| 170 | TMEM134 |  |  | 12869 | -4.067 | -0.1630 | Yes |
| 171 | TMEM16A |  |  | 12927 | -4.253 | -0.1557 | Yes |
| 172 | HSPC152 |  |  | 13036 | -4.582 | -0.1513 | Yes |
| 173 | FIBP |  |  | 13083 | -4.770 | -0.1416 | Yes |
| 174 | SUV420H1 |  |  | 13088 | -4.780 | -0.1288 | Yes |
| 175 | P2RY2 |  |  | 13104 | -4.848 | -0.1166 | Yes |
| 176 | SCGB1D2 |  |  | 13121 | -4.913 | -0.1043 | Yes |
| 177 | TNCRNA |  |  | 13139 | -4.984 | -0.0919 | Yes |
| 178 | CCND1 |  |  | 13160 | -5.098 | -0.0793 | Yes |
| 179 | WDR71 |  |  | 13188 | -5.312 | -0.0668 | Yes |
| 180 | BBS1 |  |  | 13231 | -5.710 | -0.0543 | Yes |
| 181 | SCGB2A2 |  |  | 13251 | -5.975 | -0.0393 | Yes |
| 182 | SSH3 |  |  | 13315 | -7.460 | -0.0235 | Yes |
| 183 | POLD4 |  |  | 13319 | -8.655 | 0.0001 | Yes |
Table: GSEA details [plain text format]

  

Fig 2: CHR11Q13: Random ES distribution      
 Gene set null distribution of ES for **CHR11Q13**
